# Development of Provesicular Nanodelivery System of Curcumin as a Safe and Effective Antiviral Agent: Statistical Optimization, In Vitro Characterization, and Antiviral Effectiveness

**DOI:** 10.3390/molecules25235668

**Published:** 2020-12-01

**Authors:** Farid A. Badria, Abdelaziz E. Abdelaziz, Amira H. Hassan, Abdullah A. Elgazar, Eman A. Mazyed

**Affiliations:** 1 Department of Pharmacognosy, Faculty of Pharmacy, Mansoura University, Mansoura 35516, Egypt; badri002@mans.edu.eg; 2 Department of Pharmaceutical Technology, Faculty of Pharmacy, Kafrelsheikh University, Kafrelsheikh 33516, Egypt; Abdelaziz_abdrabo@pharm.kfs.edu.eg; 3 Department of Pharmaceutics, Faculty of Pharmacy, Beni-Suef University, Beni-Suef 62511, Egypt; amira.abdelatef@pharm.bsu.edu.eg; 4 Department of Pharmacognosy, Faculty of Pharmacy, Kafrelsheikh University, Kafrelsheikh 33516, Egypt; Abdulah.elgazar@phr.mans.edu.eg

**Keywords:** Curcumin, proniosomes, optimization, molecular docking, antiviral activity

## Abstract

Curcumin is a natural compound that has many medical applications. However, its low solubility and poor stability could impede its clinical applications. The present study aimed to formulate dry proniosomes to overcome these pitfalls and improve the therapeutic efficacy of Curcumin. Curcumin-loaded proniosomes were fabricated by the slurry method according to 3^2^ factorial design using Design-Expert software to demonstrate the impact of different independent variables on entrapment efficiency (EE%) and % drug released after 12 h (Q_12h_). The optimized formula (F5) was selected according to the desirability criteria. F5 exhibited good flowability and appeared, after reconstitution, as spherical nanovesicles with EE% of 89.94 ± 2.31% and Q_12h_ of 70.89 ± 1.62%. F5 demonstrated higher stability and a significant enhancement of Q_12h_ than the corresponding niosomes. The docking study investigated the ability of Curcumin to bind effectively with the active site of DNA polymerase of Herpes simplex virus (HSV). The antiviral activity and the safety of F5 were significantly higher than Curcumin. F5 improved the safety of Acyclovir (ACV) and reduced its effective dose that produced a 100% reduction of viral plaques. Proniosomes could be promising stable carriers of Curcumin to be used as a safe and efficient antiviral agent.

## 1. Introduction

Herpes simplex virus (HSV) is a highly contagious and endemic pathogen that causes herpes labialis, herpes genitalis, encephalitis, and keratitis [1]. HS viral infections are considered to be a significant health problem worldwide [2]. The HSV infection caused by both type 1 (HS-1) and type 2 (HS-2) viruses could be transmitted through close personal contact [3]. The clinical use of antiviral agents such as ACV, foscarnet, and ganciclovir results in severe adverse effects and the development of drug-resistant viruses that impede their long-term administration [4]. Hence, there is an urgent need for developing less toxic, cheap, and readily available, alternate antiviral agents for the treatment of HSV infection. Medicinal plants offer an effective alternative because of their safety, availability, and promising therapeutic effect [5].

Turmeric (*Curcuma longa* Linn) is generally recognized as safe (GRAS) by the FDA [6] and is extensively used as a folk medicine for its antioxidant, anti-inflammatory, and antiseptic effects, particularly in Asian countries [7]. Curcumin (1,7-bis(4-hydroxy-3-methoxyphenyl)-1,6-heptadiene-3,5-dione) is the main curcuminoid derived from turmeric rhizomes [2,8]. The therapeutic efficacy and safety of curcumin (up to 12 g/day) make it an attractive target for the prevention and treatment of different human diseases [9]. Curcumin has exhibited multiple clinical applications including anti-oxidant, anti-cancer, anti-inflammatory, anti-rheumatic, anti-microbial, and hepatoprotective effects [10]. Curcumin has also been investigated for its antiviral activity against a broad spectrum of viruses such as HSV, hepatitis virus, Zikavirus, Influenza virus, Adenovirus and coronavirus (COVID19) [11,12,13,14]. The antiviral activity of Curcumin against HSV could be attributed to interference with the metabolism of the virus through cell signaling and apoptosis [12,13,15]. However, the therapeutic applications of Curcumin have been hindered by its low aqueous solubility, low permeability, poor stability, and rapid in vivo degradation [16].

Nanotechnology is a rational approach that could overcome these pitfalls due to its ability to enhance the therapeutic efficacy of drugs and minimize their adverse effects by prolonging drug release through encapsulating drug within the vesicular carriers that can cross cell membranes [17]. Additionally, nanotechnology can improve the delivery and efficacy of antiviral drugs via targeting them to their specific sites of action [18].

Liposomes are attractive phospholipid-based vehicles that could encapsulate both hydrophilic and hydrophobic drugs. Nevertheless, the physical and chemical instability is the major limitation for the widespread application of this drug delivery system [19]. Niosomes are nanovesicles composed of non-ionic surfactants. They are more stable and less expensive alternative to liposomes. However, niosomes still have some physical instability shortcomings such as fusion, aggregation, and leaking problems [20].

Proniosomes are the provesicular form of niosomes that could be prepared as free-flowing powder in which the non-ionic surfactant is coated onto a hydrophilic carrier or as a liquid crystalline gel. Both types are converted into niosomal dispersion through reconstitution with water prior to use. There are various advantages of proniosomes over niosomes, including superior physical stability, protection of the entrapped drug from hydrolysis and higher convenience of transportation and storage [21]. Other researchers [22] studied the topical administration of Curcumin via formulation as a proniosomal gel. The current work investigated the formulation of dry proniosomal powder which is considered a promising platform that could provide unit dosing of drug in addition to improving its stability and solubility. Moreover, upon the addition of water, a niosomal suspension is formed which is suitable for administration by oral or other routes [23]. Besides, dry proniosomes are a free-flowing powder that can be further processed as beads, capsules, or tablets [24].

Molecular docking is an important strategy that could explain and predict the possible therapeutic effects of many drugs [25]. The molecular docking and virtual screening can mimic the actual biological systems [26] and could predict how two or more molecular structures (drug and protein or enzyme) interact together [27].

The present study seeks to formulate dry proniosomes of Curcumin as a controlled drug delivery carrier in order to improve the solubility and the stability of Curcumin in addition to investigating their cytotoxicity and antiviral activity in the treatment of HSV-1, which is supported by the molecular docking study.

## 2. Materials and Methods

### 2.1. Materials

Curcumin, Polyoxyethylene (20) sorbitan monooleate (Tween 80), and cholesterol (CHOL) were obtained from Sigma Chemical Co. (St. Louis, MO, USA). Sorbitan monostearate (Span 60) was purchased from Oxford Lab Chemicals (Mumbai, India). Potassium monohydrogen phosphate and potassium dihydrogen phosphate were obtained from Alpha Chemica (Mumbai, India). Chloroform was obtained from El-Nasr Pharmaceutical Chemical Company (Cairo, Egypt). Spectra/Pore^®^ dialysis membranes (Spectra/pore 4, 12,000–14,000 Mwt cut-off, diameter 16 mm) were purchased from Spectrum Laboratories Inc. (Rancho Dominguez, CA, USA). HS-1 virus was kindly provided by Dr. R.G. Hughes (Roswell Park Memorial Institute, Buffalo, New York, NY, USA). *Vero* cells (African green monkey kidney cells, *Vero* C1008 [Vero 76, clone E6, Vero E6]) were purchased from Viromed Laboratories (Minnetonka, MN, USA). Dulbecco’s modified Eagle’s medium (DMEM), Penicillin G, and streptomycin sulfate were purchased from Sigma Chemical Co. (St. Louis, MO, USA). Calf serum was obtained from HyClone Laboratories, Inc. (Logan, UT, USA). All other chemicals and solvents were of analytical grade and used as received.

### 2.2. Methods

#### 2.2.1. HPLC Assay of Curcumin

A validated HPLC procedure was performed using the Dionex UltiMate 3000RS HPLC system (Thermo ScientificTM, DionexTM, Sunnyvale, CA, USA) [10]. Chromeleon 7 was the software used for collecting and processing data. The Curcumin samples (20 μL) were injected onto an Inertsil reversed-phase C18 (250 × 4.6 mm) column. The mobile phase was acetonitrile–0.1% acetic acid at a ratio of (60:40, *v*/*v*%). The samples were filtered through nylon membrane filters (Nylon Acrodisc, Gelman Sciences Inc., Ann Arbor, MI, USA). The elution was done at a flow rate of 1 mL/min. All measurements were done at room temperature. The wavelength of maximum absorption of Curcumin was 427 nm.

#### 2.2.2. Preliminary Screening Studies

The preliminary screening test was performed in order to select the proper hydration volume and the optimal hydration time for the reconstitution of proniosomes. Curcumin-loaded proniosomes have been prepared using Span 60 and 150:100 surfactant to CHOL µmolar ratio. The proniosomes were reconstituted using different hydration volumes (10 and 20 mL) for different hydration times (5 and 30 min). The prepared proniosomal formulations were evaluated for their EE% that describes the integrity of the lipid bilayer and the absence of drug leakage.

#### 2.2.3. Preparation of Curcumin-Loaded Proniosomes

Curcumin-loaded proniosomes were prepared by the slurry method [28,29]. Curcumin (50 mg), the non-ionic surfactant and CHOL were dissolved in 10 mL chloroform. The slurry was formed by introducing the lipid mixture into a 100-mL round-bottom flask containing 250 mg maltodextirn. The organic solvent was evaporated using a rotary evaporator (Buchi rotavapor R-3000, Flawil, Switzerland) that was adjusted to 100 rpm at 60 ± 2 °C under reduced pressure of 16 mm Hg. The evaporation process was continued until the content in the round-bottom flask becomes completely dry powder. The Curcumin-loaded proniosomes were stored in a tightly closed container to be used for further studies.

#### 2.2.4. Experimental Design

Nine Curcumin proniosomes were optimized according to a 3^2^ factorial design using Design-Expert software, Version 7.0.0 (Stat-Ease, Inc., Minneapolis, MN, USA) to demonstrate the impact of the independent variables on different responses [30]. The ratio of surfactant to CHO and type of surfactant were considered as the independent variables X1 and X2, respectively. The entrapment efficiency (EE%, Y1) and the percentage of Curcumin released after 12 h (Q_12h_, Y2) were selected as the dependent variables. Each factor was screened at three levels (−1, 0, and +1) that labeled the lower, the middle, and the upper levels, respectively.

The coefficient of determination (R^2^), predicted R^2^ and adjusted R^2^ were estimated in addition to plotting diagnostic plots for both EE% and Q_12h_ in order to demonstrate the goodness of fit of the present model to the experimental results. The statistical analysis of the data was performed by the analysis of variance (ANOVA) for estimation of the significance level of each term according to the *p*-value and F-statistics [31].

#### 2.2.5. Preparation of Proniosome-Derived Niosomal Dispersions of Curcumin

The niosomal dispersion of Curcumin was fabricated by hydrating the previously prepared proniosomal powder using phosphate buffer (pH = 7.4) at 60 °C ± 2 °C (The time and volume of hydration were determined according to the preliminary test). The dispersion was agitated using a vortex mixer (BOECO, Hamburg, Germany) and then sonicated for 5 min using a bath sonicator (Elmasonic E 30 H, Elma, Singen, Germany). The resultant niosomal dispersion was left overnight at 4 °C in a refrigerator for complete maturation to be used for further studies [32].

#### 2.2.6. In Vitro Characterization of Curcumin-Loaded Proniosomes

##### Determination of Drug Content and EE% of Curcumin-Loaded Proniosomes

EE% of Curcumin-loaded proniosomes was estimated by the indirect method. The free (un-entrapped) Curcumin was separated by the ultracentrifugation method. A 1-mL aliquot of proniosome-derived niosomal dispersions of Curcumin was centrifuged using a cooling ultracentrifuge at 4 °C (Biofuge, primo Heraeus, Burladingen, Germany) at 14,000 rpm for 1 h [33]. The supernatant was filtered through a 0.20-µm nylon membrane filter (Nylon Acrodisc, Gelman Sciences Inc., Ann Arbor, MI, USA) and analyzed for its drug content using HPLC at 427 nm. Encapsulation efficiency was calculated as follows:

(1) EE(%) = (Ci−Cs) × 100/C

where Ci  =  Initial amount of Curcumin, Cs  =  Amount of Curcumin in the supernatant.

The total drug content of Curcumin (unentrapped + entrapped) was determined by disrupting 1 mL of the proniosome-derived niosomal dispersion with 100 mL isopropyl alcohol [34]. The samples were filtered using a 0.20 µm nylon membrane filter (Nylon Acrodisc, Gelman Sciences Inc., USA) and analyzed for drug content using HPLC at 427 nm.

##### In Vitro Release Study of Curcumin-Loaded Proniosomes

The in vitro release study of Curcumin-loaded proniosomes was demonstrated by the dialysis method using a glass cylinder which is attached to the dissolution apparatus shaft (USP apparatus II, Erweka DT-720, Kreuzau, Germany) [17,35,36]. Initially, the semi-permeable cellulose membrane was hydrated using a phosphate buffer solution of pH = 7.4 [10] at 25 °C for 24 h. To maintain the sink condition, 0.5% (*w*/*v*) SLS was added to phosphate buffer, pH = 7.4, and used as the dissolution medium [37]. The receptor chamber contained 100 mL of dissolution medium which was kept at 37 ± 0.5 °C, to simulate the in vivo conditions, and stirred at 50 rpm. The prehydrated cellulose membrane was mounted between the donor and receptor compartments. 1 mL of the proniosome-derived niosomal dispersion containing entrapped Curcumin was placed over the cellulose membrane in the donor chamber. A 200-µL aliquot was withdrawn at different time intervals and replenished by the same volume of fresh buffer solution to retain a constant volume of the receptor medium [30]. Triplicate measurements were performed and the withdrawn samples were filtered using a 0.20-µm membrane filter and analyzed for their drug content by HPLC at 427 nm. The data are expressed as mean % Curcumin released ± SD. The release profiles of the reconstituted proniosomal formulations were plotted by taking the % Curcumin released as the Y-axis and time as the X-axis.

Different mathematical models (zero-order, first-order, the Higuchi diffusion model, Korsmeyer–Pappas, and Hixson Crowell equation) were used to describe the mechanism of in vitro release and the proper kinetic model of Curcumin–loaded proniosomes. The highest coefficient of determination value (R^2^) denoted the order of in vitro drug release [38].

##### Statistical Optimization of Curcumin-Loaded Proniosomes

The optimized Curcumin-loaded proniosomal formula was determined on the basis of the desirability function that describes the closeness of different responses to their optimal values [39]. The optimized formula was chosen on the basis of maximum EE% and maximum Q_12h_. The formulation that has the highest desirability value is selected as the optimized formula because it has the most desirable responses. The optimized formula was also validated by calculation of % relative error by comparing the observed values of different responses, %EE and Q_12h,_ with their predicted values as follows [40]:

(2) $$\%\;\mathrm{Relative}\;\mathrm{error}=\;\frac{\left(\mathrm{predicted}\;\mathrm{value}-\mathrm{observed}\;\mathrm{value}\right)\times 100\;}{\mathrm{predicted}\;\mathrm{value}}$$

The optimized Curcumin-loaded proniosomal formula was subjected to further characterization tests.

#### 2.2.7. Comparative Study of the Optimized Curcumin-Loaded Proniosomal Formula and the Conventional Niosomes

A comparative study was performed between the optimized Curcumin-loaded proniosomal formula and the corresponding niosomes by determination of EE%, Q_12h_ and stability test.

##### Formulation of Curcumin-Loaded Niosomes

The thin-film hydration method [41] was used for formulating the corresponding niosomal dispersion. Briefly, Curcumin (50 mg), the non-ionic surfactant and CHOL, at the chosen concentrations, were dissolved in chloroform (10 mL) in a round bottom flask. Chloroform was then evaporated at 60 ± 2 °C under reduced pressure of 16 mm Hg using a rotary evaporator (Buchi rotavapor R-3000, Flawil, Switzerland) forming a thin lipid film on the flask walls. The thin film was rehydrated by phosphate buffer, pH 7.4 (10 mL) at 60 ± 2 °C for 30 min. The niosomal dispersion was agitated using a vortex mixer (BOECO, Hamburg, Germany) and then sonicated for 5 min using a bath sonicator (Elmasonic E 30 H, Elma, Singen, Germany). The niosomal dispersion was stored overnight at 4 °C for complete maturation.

##### Evaluation of EE% and In Vitro Release of Curcumin-Loaded Niosomes

Both EE% and the in vitro release of Curcumin-loaded niosomes were tested as previously described.

##### The Stability Test

The stability test was used to study the impact of storage on the optimized Curcumin-loaded proniosomal formula and the corresponding niosomes. Both formulations were placed in a tightly closed glass vial at (4–8 °C) for three months [42]. The formulations were evaluated with regard to their drug content, EE%, and Q_12h_. The significance of difference, between the fresh and the stored formulations, was estimated by Student’s t-test using SPSS-11 software (SPSS. Inc., Chicago, IL, USA).

Moreover, the in vitro release profile of the stored formulations was compared to that of the fresh preparations by the similarity factor test [38,43]. The f2 was calculated according to the following equation:

(3) $$F2=50.\log\left\{{\left[1+\frac{1}{n}\overset{n}{\underset{t=1}{\sum }}{\left(\mathrm{Rt}-\mathrm{Tt}\right)}^{2}\right]}^{-0.5}\right\}100$$

where n is the number of sampling points and R_t_ and T_t_ are the % Curcumin released from the fresh and the stored formula, respectively, at time t. If the f_2_ value lies between 50 and 100, the two release profiles are considered to be similar.

#### 2.2.8. Characterization of the Optimized Curcumin-Loaded Proniosomes

##### Scanning Electron Microscopy (SEM)

The morphology of the reconstituted Curcumin-loaded proniosomal formula was investigated using a scanning electron microscope (JSM 6100 JEOL, Tokyo, Japan). 0.1 mL of the optimized formula was diluted by 10 mL of deionized water. One drop of the diluted Curcumin-loaded niosomal dispersion was placed onto a SEM sample stub using a carbon adhesive tape. Afterward, the sample was and dried and scanned by SEM [44].

##### Vesicle size and Zeta Potential Determination

The size and zeta potential of the Curcumin-loaded nanovesicles were determined for the investigation of the colloidal characteristics of the reconstituted proniosomal formula. The reconstituted proniosomal formula (0.1 mL) was properly diluted with deionized water (10 mL). The NICOMP 380 ZLS zeta potential/particle sizer (PSS Nicomp, Santa Barbara, CA, USA), with a scattering angle of 90°, was then used to estimate vesicle size and zeta potential [45] at 25 °C. Triplicate measurements were performed.

##### Determination of the Micromeritic Properties

The micromeritic properties of the optimized proniosomal powder and maltodextrin were determined by calculating the angle of repose of powders using the funnel method. The tested powders were poured through a funnel so that its outlet orifice is 5 cm above the surface of a graph paper. The powders flowed down forming a cone on the graph paper [46]. The angle of repose was estimated as follows:

(4) Tan θ=h/r 

(θ is the angle of repose, h is the height of the powder cone, and r is the radius of the base of powder cone).

#### 2.2.9. Evaluation of the Antiviral Activity and Cytotoxicity

The antiviral activity of the reconstituted proniosomal formula was tested against the HS-1 virus which was grown on *Vero* (African green monkey kidney) cells. The cytotoxicity and antiviral activity of the proniosomal formula were compared with that of the corresponding Curcumin dispersion. Additionally, the antiviral activity and cytotoxicity of ACV dispersion were studied in the presence and absence of the optimized proniosomal formula.

##### HS-1 Virus and the Cell Culture

HS-1 virus stocks were prepared as culture medium aliquots from *Vero* cells infected with a multiplicity of 1 virion/10 cells and cultured for 3 days and stored at (−80 °C) until used. Preparing the working stocks was performed by titering virus through serial dilution in the culture medium. They were assayed, in triplicate, on *Vero* monolayers in the wells of microtiter trays (Falcon Microtest III 96-wells trays, Becton Dickinson Labware, Lincoln Park, NJ, USA) [47].

*Vero* cells were grown in DMEM supplemented with 10% *v*/*v* calf serum, Penicillin G (60 μg/mL) and streptomycin sulfate (100 μg/mL) and kept in a humidified atmosphere containing CO_2_ (15% *v*/*v*) in air at 37 °C. *Vero* stocks were maintained in culture flasks containing medium supplemented with 1% *v*/*v* calf serum at 34 °C. Subcultures, for both antiviral screening and virus titration, were grown in the wells of microtiter trays through suspending *Vero* cells in medium following trypsin-EDTA treatment. That was followed by counting the suspension using a hemocytometer, diluting in medium containing 10% *v*/*v* calf serum to 2 × 10^4^ cells/200 mL culture, aliquoting into different wells of the tray, and finally culturing until reaching confluency [48].

##### Determination of the Antiviral Activity

The antiviral activity was studied using an improved plaque-reduction assay [4,49]. Microtiter trays having confluent monolayer cultures of *Vero* cells were inverted. The medium was shaken out and then replaced with serial dilutions of the samples (in triplicate) in a 100-µL medium followed by titering virus into (100-µL medium containing 10% *v*/*v* calf serum) in different wells. The last row of wells was kept for the control that was not treated with samples or HS-1 virus. Different trays were cultured for 66 h. After the appropriate incubation period, the trays were inverted on pads of paper towels. The remaining cells were rinsed well using medium and fixed, for about 20 min, using formaldehyde (3.7% *v*/*v*) in saline and stained with crystal violet (0.4%). The improved plaque-reduction assay investigated the efficacy of the tested agents on the inhibition of HS-1 viral infection of *Vero* cells via determination of the % reduction in the number of viral plaques when compared with the untreated group. The viral particles which are not neutralized by the tested agents would infect the cells forming a plaque. The cytotoxicity was assessed as the concentration that achieved a fifty percent loss of the monolayer present around the plaques caused by HS-1 virus (CC_50_) [47].

#### 2.2.10. Statistical Analysis

Statistical analysis was conducted by one-way ANOVA and the Student’s t-test using SPSS-11 software (SPSS. Inc., Chicago, IL, USA). Investigating the impact of different independent variables on EE% and Q_12h_ of the Curcumin-loaded proniosomal formulations was performed by ANOVA using Design-Expert software, Version 7.0.0 (Stat-Ease, Inc., Minneapolis, MN, USA). A *p*-value lower than 0.05 describes statistically significant differences.

## 3. Results and Discussion

### 3.1. Preliminary Screening Studies

The preliminary screening test is an important tool that is used for the determination of the most suitable parameters for the pharmaceutical formulation. The reconstitution of proniosomes into niosomes is a vital factor that could affect the encapsulation efficiency of the proniosomal vesicles [34]. Therefore, both the time and the volume of hydration were studied to select the proper conditions for the reconstitution of proniosomes (Table 1). The results investigated that the volume of the hydration had a significant negative impact (*p* < 0.05) on EE%. These findings could be attributable to increased leakage of the drug from niosomal vesicles at larger hydration volume [34]. However, the time of hydration had no significant effect (*p* > 0.05) on %EE. These results are in accordance with Pawar et al. who reported that there was no significant influence of the time of hydration on the EE% of niosomal vesicles [50]. Therefore, 5 min hydration time is selected for further studies because short hydration time would improve patient acceptability and product applicability. Other researchers such as Sammour et al. [34] reported that the hydration time has a significant (*p* < 0.05) negative effect on %EE. However, Ruckmani and Sankar found that more drug would be entrapped within the vesicles at longer hydration time [51].

According to the preliminary screening test, 10 mL hydration volume and 5 min hydration time were selected for the reconstitution of proniosomes.

### 3.2. Formulation of Curcumin-Loaded Proniosomes

Curcumin-loaded proniosomes have been prepared using the slurry method. The main constituents of proniosomes are a surfactant, a hydrophilic carrier, and a membrane stabilizer. The involved excipients are FDA approved excipients [52] and generally recommended as safe (GRAS) [53]. Span 60, Tween 80, and a mixture of the two surfactants have been used as the non-ionic surfactants. Non-ionic surfactants are used in the preparation of the proniosomal vesicles due to their safety, compatibility and stability [54]. CHOL has been added as a membrane stabilizer of the nanovesicles. Its addition improves the stability of the lipid bilayer by increasing the gel liquid transition temperature of the vesicle [17]. Hydrophilic carriers increase the surface area, impart flexibility in the ratio of surfactant and other constituents, and provide more efficient loading [55]. Maltodextrin was selected as the coating carrier due to its safety and good aqueous solubility for ease of reconstitution. Moreover, maltodextrin has poor solubility in chloroform that permits easy coating of the maltodextrin particles after adding the surfactant solution and then evaporating the solvent [56].

### 3.3. Reconstitution of Curcumin-Loaded Proniosomes into Niosomes

Curcumin-loaded proniosomes were reconstituted by hydrating the proniosomal powder with phosphate buffer (pH = 7.4) at 60 ± 2 °C [57]. The addition of water results in swelling of the lipid bilayer due to the interaction between the hydrophilic groups of the surfactants and water leading to the development of multilamellar and spherical shaped vesicles [17]. The multi-lamellar vesicles are further transformed into unilamellar niosomal vesicles by sonication [58].

The formulation of niosomal dispersions by the reconstitution of proniosomal powder is more convenient than other conventional methods for preparing niosomes. That could be explained on the basis of the higher stability of the proniosomal powder compared to the corresponding niosomes [59]. Moreover, coating the surfactant film on the carrier surface, instead of the inner wall of the round bottom flask, results in the formation of a thinner film onto a wider surface. Therefore, niosomal vesicles, with high drug loading capacity, are developed through easy hydration of this film [60].

The preparation of niosomes via standard techniques results in the formation of unstable heterogeneous dispersion that is prone to aggregation or sedimentation. Additionally, the complete hydration of the lipid film is difficult even after vigorous shaking. Sometimes, a lipid residue remains on the round bottom flask wall, and additional time is needed. Upon hydration, this layer becomes more viscous and tends to remain adhered to the flask wall. As a result, the loss of some lipids during the formulation process is possible [61].

Other researchers such as Abd-Elbary et al. [62] compared the characteristics of the reconstituted proniosomes with the corresponding niosomal vesicles. The conventional niosomes were found to be larger and more heterogeneous than reconstituted proniosomes.

### 3.4. Analysis of the 3^2^ Factorial Design

The objective of the optimization process is to explore the effects of different variables on the characteristics of the formulation and to determine the levels of variables required for the development of high-quality products [63]. For the optimization of Curcumin-loaded proniosomes, a 3^2^ factorial design was used (Table 2). Two independent variables have been chosen: the ratio of surfactant to CHOL (X1) and the type of surfactant (X2). The selection of the optimized proniosomal formula was on the basis of maximizing both EE% (Y1) and Q_12h_ (Y2).

Table 3 summarizes the output data of the factorial design of Curcumin-loaded proniosomes. The adequate precision measures the signal to noise ratio. In this model, adequate precision values of both responses were more than 4 (the desired value) demonstrating that the model is capable of navigating the design space. Data of both responses (Y1 and Y2) provided a reasonable fit to the linear model (R^2^ = 0.9676 and 0.9822, respectively). The relatively high values of R^2^, predicted R^2^, and adjusted R^2^ of both responses showed that the obtained equations are statistically valid and fit to the obtained results. The predicted R^2^ estimates the response value predictability of the model. In addition, the predicted and adjusted R^2^ values are in reasonable agreement, because the difference between them is lower than 0.20 [64].

**Figure 1:**
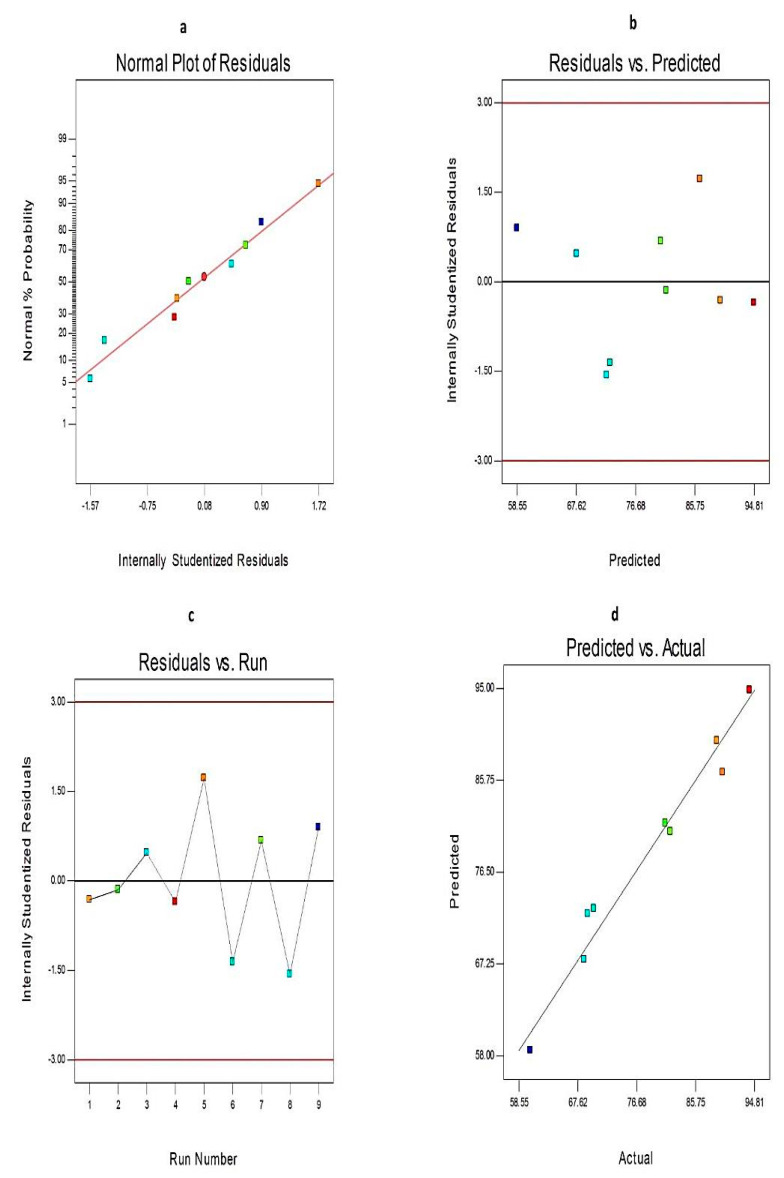
The diagnostic plots for entrapment efficiency (EE)% of Curcumin-loaded proniosomes (**a**) normal probability plot (**b**). internally studentized residuals versus predicted values plot, (**c**) internally studentized residuals versus run number plot and (**d**) predicted versus actual values plot. Abbreviation: EE, entrapment efficiency.

**Figure 2:**
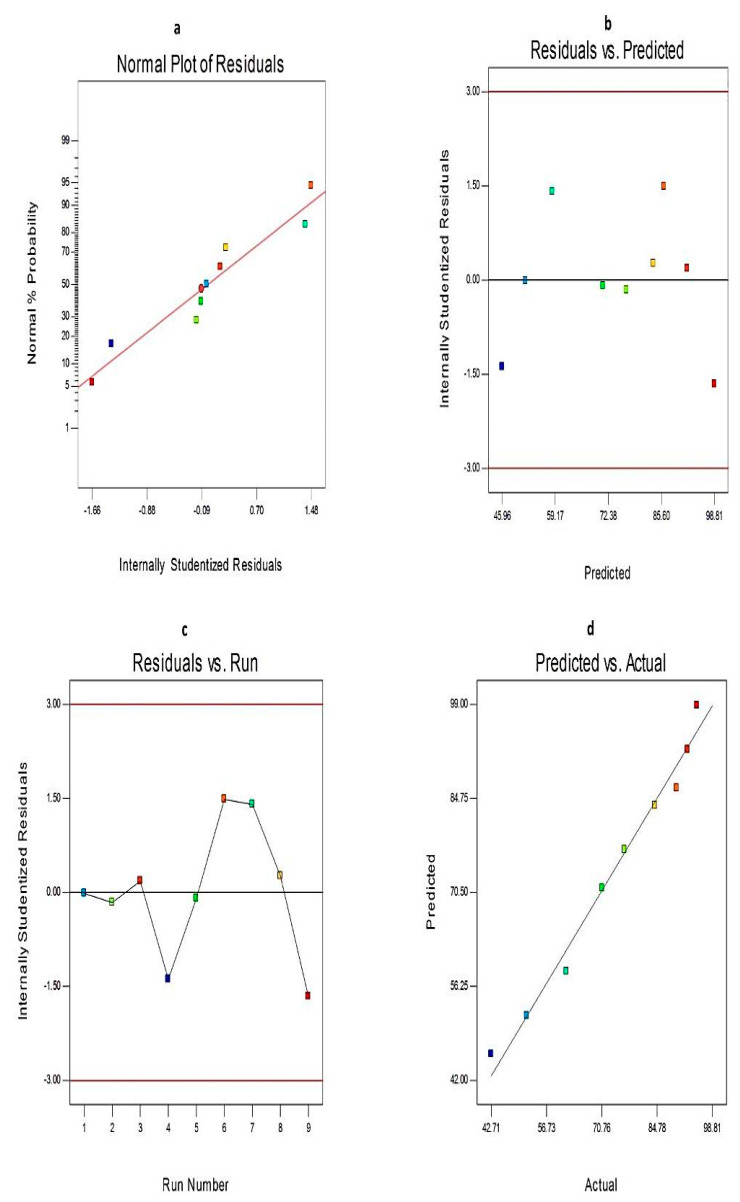
The diagnostic plots for Q_12h_ of Curcumin-loaded proniosomes; (**a**) normal probability plot, (**b**) internally studentized residuals versus predicted values plot, (**c**) internally studentized residuals versus run number plot, and (**d**) predicted versus actual values plot. Abbreviation: Q_12h_, % drug released after 12 h.

Besides, diagnostic plots for both EE% and Q_12h_ were developed to ensure the goodness of fit of the present model and to assess its credibility, Figure 1 and Figure 2, respectively. The residuals were computed as the difference between the actual and predicted values of the responses. The normal probability plots of residuals, Figure 1a and Figure 2a, demonstrate a linear pattern with a normal distribution of the residuals, and hence, the data need no transformation. A plot of the residuals versus the predicted responses, Figure 1b and Figure 2b, exhibit that the color points depicting the values of both responses were scattered randomly and located within the limits near to the zero-axis investigating the absence of constant error. Figure 1c and Figure 2c represent the residual versus run plots and describe a uniform and random scattering of points, showing that no lurking variables affect the studied responses. Figure 1d and Figure 2d investigated the excellent analogy between the observed and the predicted values of Y1 and Y2 of Curcumin-loaded proniosomes in the current model.

ANOVA study demonstrates the significance of the influence of different independent variables on both responses (Y1 and Y2). When the *p*-value is less than 0.05, the null hypothesis (H_0_) is dismissed and the alternate hypothesis is accepted (Table 4).

#### 3.4.1. The Effect of Formulation Variables on EE% of Curcumin-Loaded Proniosomes

**Figure 3:**
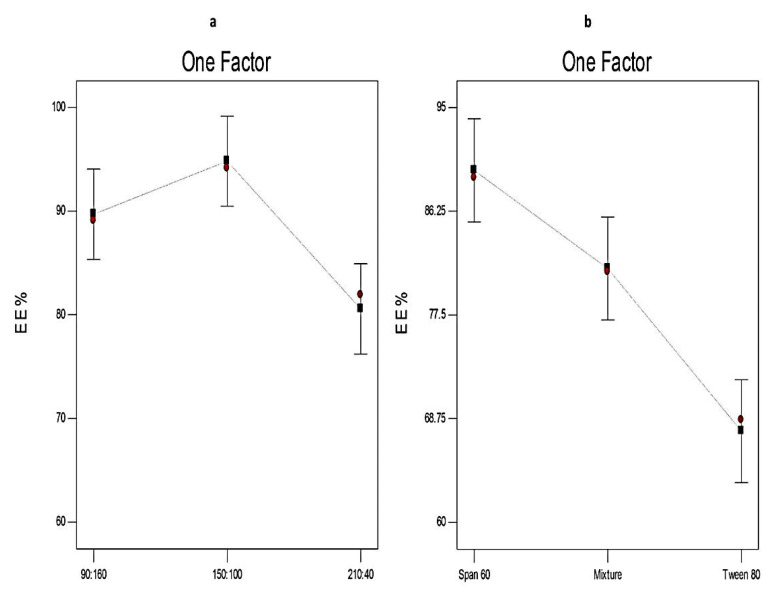
The effect of different independent variables (**a**) ratio of surfactant to cholesterol (CHOL) and (**b**) type of surfactant on EE% of Curcumin-loaded proniosomes according to 3^2^ factorial design. Abbreviation: CHOL, cholesterol; EE, entrapment efficiency.

The drug content (entrapped + un-entrapped) of Curcumin-loaded proniosomes ranged from 95.26 ± 1.33 to 103.56 ± 1.69%. Table 2 demonstrates that the EE% of Curcumin-loaded proniosomes ranged from 60.33 ± 1.61 to 94.11 ± 1.27%. Figure 3 investigates the impact of different variables on the EE% of Curcumin-loaded proniosomes. The statistical analysis (Table 4) confirms that both the ratio of surfactant to CHOL and the type of surfactant have a significant influence on %EE.

Concerning the ratio of surfactant to CHOL (X1), it is clear that increasing the CHOL concentration has a significant impact on the EE% of Curcumin-loaded proniosomes (*p* < 0.05). EE% increased significantly when the amount of CHOL increased from 40 mg to 100 mg. That may be attributable to the fact that CHOL could increase the rigidity of the lipid bilayer by acting as a vesicular cement [51]. However, the EE% decreased with further increase in CHOL concentration. This might be due to its competition with the drug for the packing space within the lipid bilayer and disrupting the regular structure of the lipid membrane. These outcomes were in agreement with El-Laithy et al. [65] and Sambhakar et al. [42] who reported that increasing the concentration of CHOL in the proniosomal formulations facilitates the development of less leaky bilayers due to its intercalation between the non-ionic surfactants bilayers. Conversely, a further increase in CHOL concentrations results in decreasing the %EE.

With respect to the type of surfactant (X2), it is obvious that X2 had a significant impact on %EE (*p* < 0.01). The EE% from different formulations prepared using different types of surfactants followed the order of; Tween 80-based vesicles< mixed surfactant-based vesicles < Span 60-based vesicles. That could be attributable to the development of less leaky nanovesicles upon using Span 60 that has higher hydrophobicity (HLB 4.7) and higher phase transition temperature (53 °C) compared to Tween 80. Conversely, Tween 80 is a hydrophilic surfactant with a high HLB value (HLB 15) and contains a double bond in its alkyl chain that hinders the development of a tight membrane and leads to the formation of more leaky vesicles [51,66].

These results are in accordance with Eldeeb et al. [67] who studied the optimal conditions for the formulation of brimonidine tartrate-loaded proniosomes and found that the EE% of Span 60-based proniosomes was higher than Tween 80-based ones.

#### 3.4.2. The Effect of Formulation Variables on Q_12h_ of Curcumin-Loaded Proniosomes

**Figure 4:**
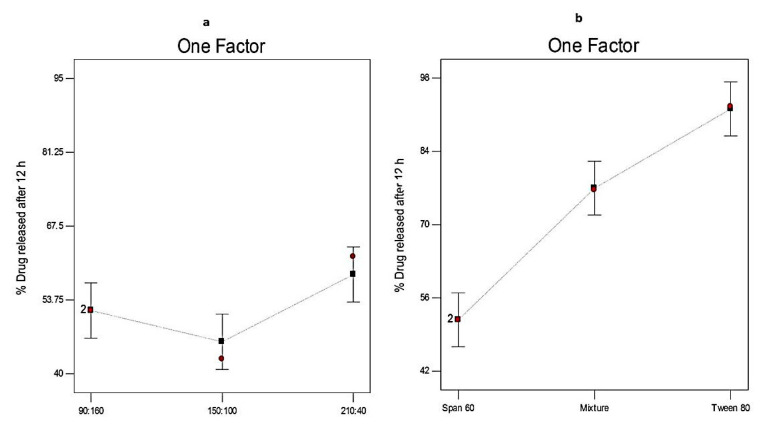
The in vitro release profile of Curcumin-loaded proniosomes and Curcumin dispersion for 12 h. (**a**) ratio of surfactant to cholesterol (CHOL) and (**b**) type of surfactant.

Figure 4 demonstrates that the Q_12h_ of different Curcumin-loaded proniosomes ranged from 42.71 ± 1.74 to 94.91 ± 2.18%. It is obvious that the release of Curcumin from different proniosomal formulations was more prolonged than the release of free drug that exhibited 93.62 ± 1.41% drug released after 4 h. These results showed that proniosomal formulations could successfully sustain the in vitro release of Curcumin because they act as a drug reservoirs [42]. Besides, the higher in vitro release profile of free Curcumin demonstrates that its in vitro release was not hampered by the semipermeable membrane and the sink condition was successfully attained [40].

**Figure 5:**
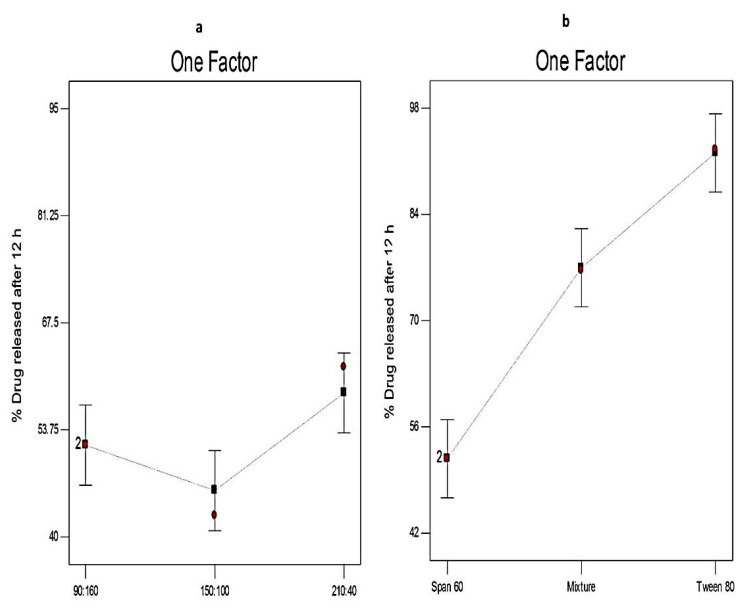
The effect of different independent variables (**a**) ratio of surfactant to CHOL and (**b**) type of surfactant on Q_12h_ of Curcumin-loaded proniosomes according to 3^2^ factorial design. Abbreviation: Q_12h_, % drug released after 12 h.

Figure 5 illustrates the influence of different independent variables on Q_12h_ (Y2). ANOVA results (Table 4) investigates that the surfactant to CHOL ratio has significant influence (*p* < 0.05) on Q_12h_. That might be due to that the in vitro release from the niosomal vesicles is dominated by the rigidity of the lipid membrane. Hence, as the concentration of CHOL increases, drug efflux decreases due to its membrane-stabilizing ability by filling the pores in the vesicular bilayer resulting in sustained drug release. However, a further increase in the concentration of CHOL reduces the leakage of the encapsulated drug by decreasing the membrane fluidity [45]. These results agreed with Shehata et al. [57] who reported that the gel-to-liquid phase transition of the vesicular membrane of proniosomes was abolished by increasing the concentration of CHOL resulting in the formation of more rigid vesicles and decreasing the drug release. However, a further increase of the CHOL concentration leads to disrupting the regular bilayer structure and increasing the efflux of the entrapped drug.

With respect to the type of surfactant, it is clear that X2 has a significant effect (*p* < 0.01) on Q_12h_ of Curcumin-loaded proniosomes. Q_12h_ in the case of Tween 80-based proniosomes was significantly higher than that from Span 60-based proniosomes. That could be explained on the basis of the higher solubilizing power of Tween 80 on hydrophobic solutes (HLB = 15) compared to Span 60 [17]. These findings are in accordance with Sambhakar et al. [42] who investigated that the in vitro release of risperidone from Span-based proniosomes was lower than that from Tween-based vesicles due to the hydrophilicity of Tweens.

According to the correlation coefficient values (R^2^), it is clear that the in vitro release profile of both Curcumin-loaded proniosomes and Curcumin dispersion followed the Higuchi model (Table 5). This kinetic pattern showed that the release of Curcumin-loaded proniosomes is dominated by the diffusion model that depends on the concentration gradient of drug between the nanovesicles and dissolution medium [68].

#### 3.4.3. The Optimization of Curcumin-Loaded Proniosomes

The optimization of Curcumin-loaded proniosomes was conducted by numerical analysis using Design-Expert software on the basis of achieving maximum EE% and maximum %drug released [69]. The selection of the optimized proniosomal formula was based on the desirability criteria.

The response variables could describe the performance of pharmaceutical formulations. In the desirability criteria, the choice of the optimized formula can be attained by the simultaneous optimization of these variables. Each response is converted into a desirability value and the total desirability value is calculated as the geometric mean of individual desirability values. The desirability value ranges from zero to one. Zero represents a completely undesirable response value and one describes a completely ideal or desirable response value. The desirability value increases as it gets closer to the target value [36,70].

F5 had the highest desirability index (0.650); hence, it was selected as the optimized formula. Moreover, the predicted values of %EE and Q_12h_ were 86.53 and 71.12%, respectively. The calculated % relative error was found to be less than 5 (−3.94 and 0.32 for Y1 and Y2, respectively). These results indicated the fitness of this model in selecting the optimized formula (F5).

### 3.5. Comparative Study of the Optimized Curcumin-Loaded Proniosomal Formula and the Conventional Niosomes

#### 3.5.1. Determination of EE%

No significant difference in %EE (*p* > 0.05) was observed between the optimized Curcumin-loaded proniosomal formula (F5) and the corresponding niosomes that have EE% of 90.78 ± 1.52%.

#### 3.5.2. In Vitro Release Study

**Figure 6:**
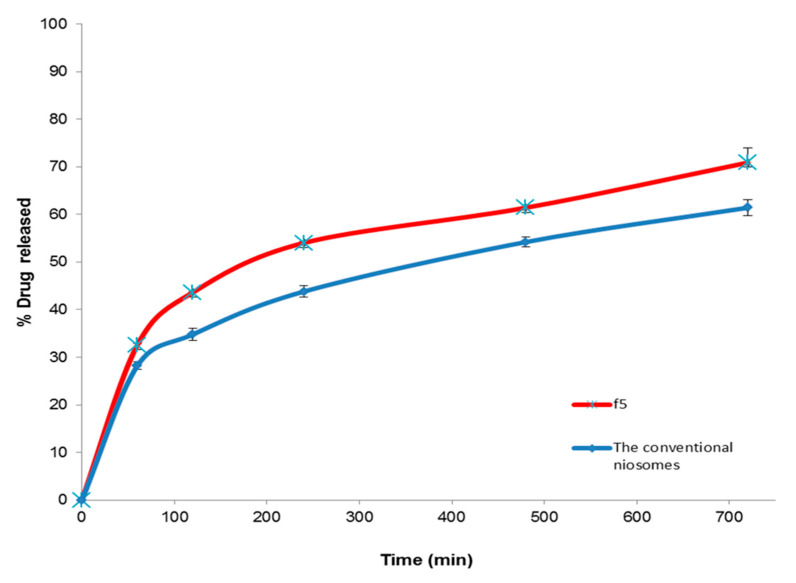
The in vitro release profile of the optimized Curcumin-loaded proniosomal formula and the conventional niosomes.

Figure 6 investigates the in vitro release profiles of Curcumin from the optimized proniosomal formula (F5) and the corresponding niosomes. It was detected that the % drug released from optimized Curcumin-loaded proniosomes was significantly (*p* < 0.01) higher than that from the conventional niosomes that demonstrated 61.42 ± 1.71% cumulative drug released after 8 h. Additionally, the similarity factor test showed that there is a significant difference between the in vitro release profiles of the two formulations because the f_2_ value was less than 50 (41). These findings may be explained on the basis of the adsorption of the lipid coat of Curcumin-loaded proniosomal vesicles on the hydrophilic carrier increasing its effective surface area [28]. Besides, it might be attributed to improving the solubility of Curcumin and changing its crystalline structure to the amorphous state after incorporation into the proniosomal vesicles [38]. Besides, Akhilesh et al. [71] reported that the in vitro release profile of proniosome-derived niosomes is superior to the corresponding niosomes due to their better size distribution. These results are also in a good agreement with Khudair et al. [72] who found that % Letrozole released from proniosomal formulations was higher than that from the conventional niosomes after 24 h.

#### 3.5.3. The Stability Study

The stability study, after storage of both F5 and the conventional niosomes for three months at 4–8 °C, explored that there was a non-significant difference (*p* > 0.05) in the drug content, EE% and Q_12h_ of the stored Curcumin proniosomal formula (F5) when compared with the fresh proniosomal formula (Table 6). Conversely, there was a significant decrease in the drug content (*p* < 0.05), EE% (*p* < 0.01) and Q_12h_ (*p* < 0.05) of the stored niosomal dispersion when compared to the fresh one. These outcomes illustrated that proniosomes are a stable drug delivery system that can solve the storage problems related to conventional niosomes. That could be attributed to formulating proniosomes as a dry powder which is more stable than the aqueous niosomal dispersion during storage.

The above findings agreed with Bhama and Sambath [73] who concluded that proniosomes provide a stable drug delivery system that could minimize the stability pitfalls associated with conventional niosomes during storage.

### 3.6. Characterization of the Optimized Curcumin-Loaded Proniosomes

The optimized Curcumin-loaded proniosomal formula (F5) was characterized with respect to its morphological properties, vesicle size, zeta potential, and micromeritic properties. Additionally, the interaction of Curcumin with different excipients and changing the crystalline structure of Curcumin within proniosomes were investigated by Fourier transform infrared spectroscopy (FTIR) [38,74,75,76,77,78] (Figure S1) and Differential scanning calorimetry (DSC) study [38,79,80,81,82,83,84,85] (Figure S2) (Supplementary information).

#### 3.6.1. Morphological Characterization by SEM

**Figure 7:**
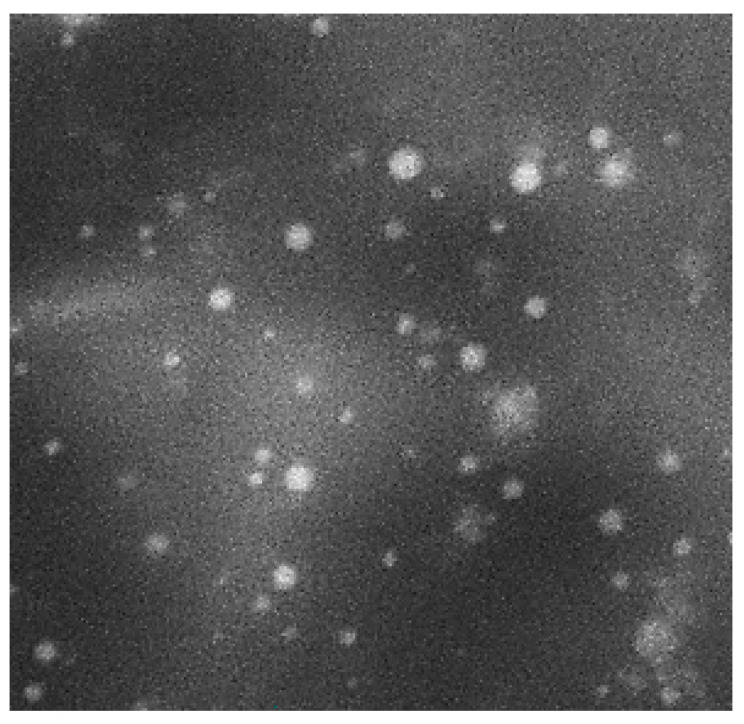
Scanning electron micrograph of the optimized Curcumin-loaded proniosomal formula (F5) after reconstitution.

Figure 7 shows the SEM micrograph of the reconstituted Curcumin-loaded proniosomes (F5) as discrete and spherical nanovesicles with sharp boundaries. The spherical shape of Curcumin-loaded niosomal vesicles may be explained on the basis of the amphoteric nature of Span 60 and Tween 80 [86] that results in the development of closed bilayer niosomal vesicles in the aqueous medium and the tendency to reduce their surface free energy forming spherical vesicles [38,87].

#### 3.6.2. Determination of Vesicle Size and Zeta Potential

**Figure 8:**
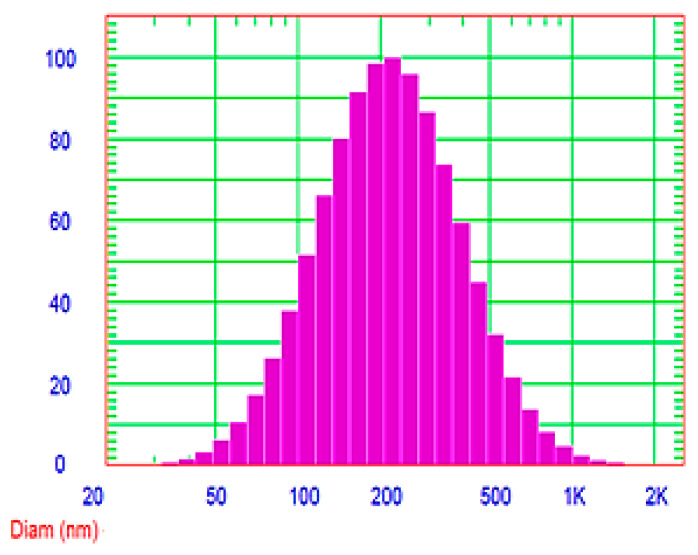
Particle size distribution curve of the optimized Curcumin-loaded proniosomal formula (F5) after reconstitution.

Figure 8 investigated the symmetric unimodal frequency distribution pattern of the optimized Curcumin-loaded proniosomal formula (F5). The vesicle size of the reconstituted proniosomal formula was 251.2 nm with a low polydispersity index (PDI) value of 0.355. A PDI value of 1 indicates a wide variation in particle size; a reported PDI value of 0 investigates the absence of the size variation [45]. The obtained small value of PDI of the reconstituted proniosomes demonstrates homogenous distribution and limited variation of the vesicle size.

**Figure 9:**
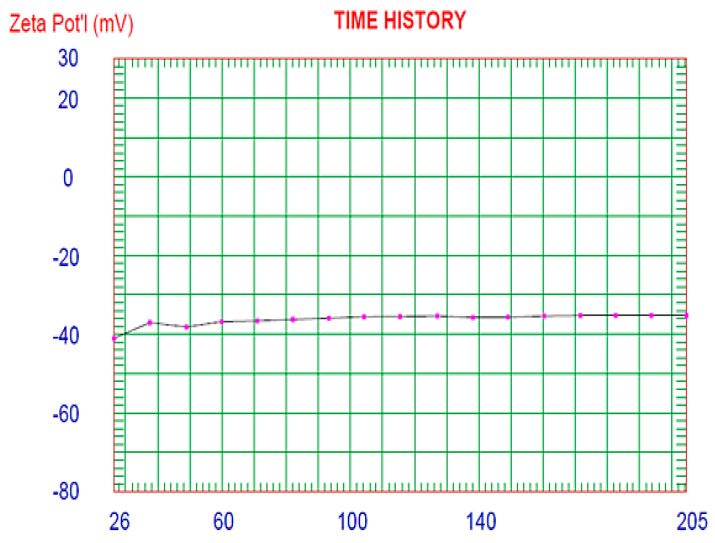
Zeta potential distribution of the optimized Curcumin-loaded proniosomal formula (F5).

Zeta potential describes the net charge of the colloidal dispersions (Figure 9). Large zeta potential value of F5 (−35.27 mv) investigates the stability of the reconstituted proniosomal dispersion due to repulsion between different niosomal vesicles as a result of the formation of a high-energy barrier between the nanovesicles that results in increasing the stability of the niosomal dispersion and preventing their agglomeration [28].

The negative zeta potential of nanovesicles, containing non-ionic surfactants, were explained by many researchers [36]. Caracciolo et al. [88] demonstrated that Tween 20-based niosomal vesicles have a negative zeta potential due to the orientation of hydroxyl groups of both Tween 20 and cholesterol with respect to water and the consequent redirection of the ionic charges in the aqueous medium. Junyaprasert et al. [89] explained the negative charge of the drug-free niosomes, containing Span 60, on the basis of the adsorption of counterions at the surface of nanovesicle. Furthermore, Pawar et al. [50] demonstrated that the ionization of free groups present on the vesicular surface could be the cause of the negative charge of Span 60-based Bifonazole niosomes. Essa et al. [90] reported that niosomes containing Span 40 have a negative zeta potential due to the preferential adsorption of hydroxyl ions on the vesicular surface.

The reasonable cellular uptake of the negatively charged nanovesicles, without repulsion with the negatively charged cell membrane, could be explained on the basis of the non-specific adsorption of nanovesicles on the cellular membrane, and consequent development of clusters of these nanoparticles [91].

#### 3.6.3. Determination of the Micromeritic Properties

Good flowability of powders has a critical role during the development of solid dosage forms. Flowability is assessed by the angle of repose (θ) which is the angle between the surface of the powder heap and the horizontal surface [92]. Studying the micromeritic properties, using the funnel method, showed that the angle of repose of Curcumin-loaded proniosomal powder (28.26° ± 0.25°) was lower than that of maltodextrin (46.24° ± 0.33°). Hence, the flowability of the optimized proniosomal powder (F5) was higher than that of maltodextrin powder.

A value of θ < 30° describes excellent powder flowability, whereas θ > 56° describes very poor flowability. The intermediate range designates good flowability (θ between 31–35°), fair flowability (θ between 36–40°), passable flowability (θ between 41–45°), and poor flowability (θ between 46–55°) [38]. Accordingly, the flowability of F5 was rated as excellent flowability, while pure maltodextrin powder has poor flowability.

These results are compatible with other studies [60,93] that investigated that proniosomes are non-sticky provesicular powders that have higher flowability than the carriers. Improving the flowability of proniosomal powder compared to the pure carrier could be attributable to the coating of the surfactant film on the carrier surface and reducing the cohesive interactions between carrier particles [60].

### 3.7. Evaluation of the Antiviral Activity and Cytotoxicity

**Figure 10:**
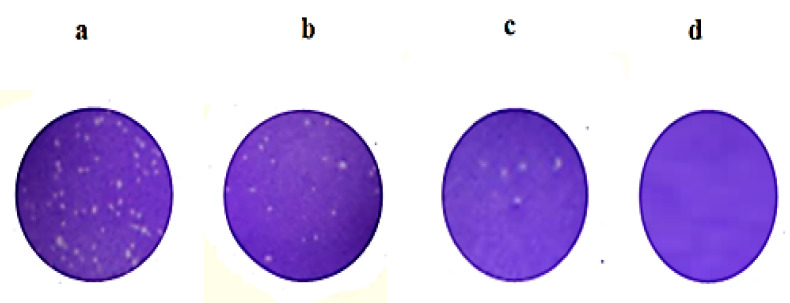
Plaque formation of Herpes simplex type 1 virus (HSV-1) for (**a**) untreated group, (**b**) Curcumin group, (**c**) F5 group, and (**d**) Curcumin and ACV group, (n = 3). Abbreviation: F5, the optimized Curcumin-loaded proniosomal formula; ACV, Acyclovir; HS-1, Herpes simplex type 1 virus.

The antiviral activity of Curcumin was demonstrated by the molecular docking study to demonstrate the ability of Curcumin to bind effectively with the active site of DNA polymerase of HSV-1 [16,94,95,96,97,98,99,100,101,102] (Figure S3) (Supplementary information). The antiviral activity of the reconstituted Curcumin-loaded proniosomal formula (F5) were tested and compared with that of Curcumin dispersion. The antiviral activity was studied using an improved plaque-reduction assay [49] against the HS-1 virus that was grown on *Vero* cells, Figure 10. This technique allows the development of HS-1 plaques without the use of thickening agents. In other techniques, the addition of thickener could cause a number of technical problems such as toxicity of the thickener to virus or host cells and interference with the tested antiviral agents [49].

The results revealed that both F5 and Curcumin were able to reduce the viral plaques by 90 ± 1.60% and 75 ± 0.82%, respectively at a concentration of 30 μM. Other researchers such as Flores et al. [2] have also reported that 30 μM Curcumin possesses the ability to serve as a therapeutic agent that minimizes the transmission of HS viruses.

It is obvious that the antiviral activity of the reconstituted proniosomal Curcumin (F5) was significantly (*p* < 0.001) higher than that of the corresponding Curcumin dispersion. The higher antiviral activity could be attributable to the interaction of the reconstituted proniosomal vesicles with viral cells by endocytosis or fusion [103,104]. The adsorption and fusion of the formed niosomal vesicles onto the membrane surface results in a high thermodynamic activity gradient of Curcumin at the interface, which is considered to be the main driving force for the permeation of lipophilic species [105]. Additionally, proniosomes can increase the permeability of Curcumin due to the penetration enhancing effect of surfactants [106]. Therefore, the proniosomal formulation could be a promising drug delivery system that could improve the antiviral activity of Curcumin. These outcomes are in agreement with Monavari et al. [104] who reported that ACV-loaded niosomes were more efficient than the free ACV as antiviral agent, because ACV-loaded niosomes showed nearly 3-fold improvement in the antiviral activity against HSV-1 than the free drug.

**Figure 11:**
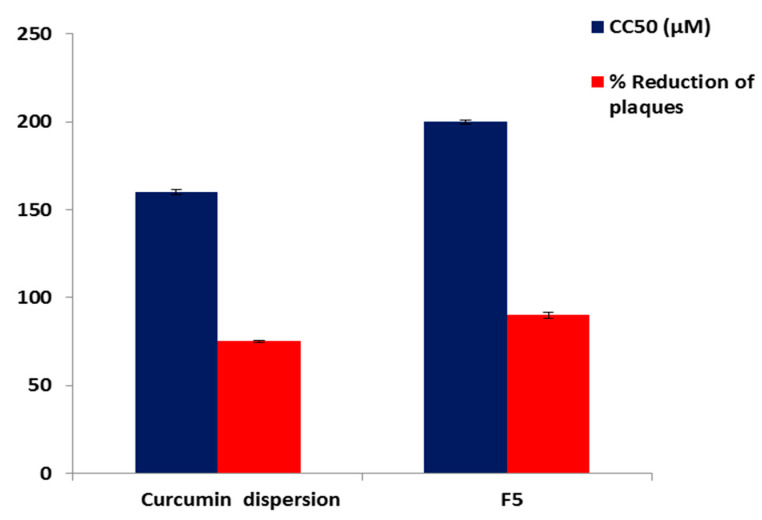
% Reduction of plaques and CC_50_ of Curcumin dispersion and F5 against HS-I virus. Each point represents the mean ± SD (n = 3). Abbreviation: F5, the optimized Curcumin-loaded proniosomal formula; HS-I, Herpes simplex type I virus; CC_50,_ the half-maximal cytotoxic concentration.

Determination of the cytotoxicity of an antiviral drug is a critical issue to ensure host safety because it should not exhibit acute or long-term host toxicity [107]. The ideal antiviral drug should have antiviral activity at very low concentrations and be cytotoxic only at very high concentrations, thus yielding high selectivity [108]. The cytotoxicity was described by the half-maximal cytotoxic concentration (CC_50_). The CC_50_ of F5 (200 μM) was significantly (*p* < 0.01 and *p* < 0.001) higher than that of Curcumin (160 μM) and ACV (80 μM), respectively (Figure 11). These findings explored the greater safety of F5 that exhibits cytotoxicity at higher concentrations than both Curcumin and ACV.

SI denotes the selectivity of the antiviral. SI was calculated by dividing CC_50_ as a measure of cytotoxicity on IC50 (50% inhibitory concentration) as a measure of inhibitory potential [109]. Interestingly, the SI value of F5 (12.5) was significantly higher (*p* < 0.01) than Curcumin dispersion (8), indicating the higher selectivity of F5.

These findings are in accordance with Anggakusuma et al. [110] who found that Curcumin nanoformulation improved the antiviral activity against the hepatitis C virus without any toxic effect.

**Figure 12:**
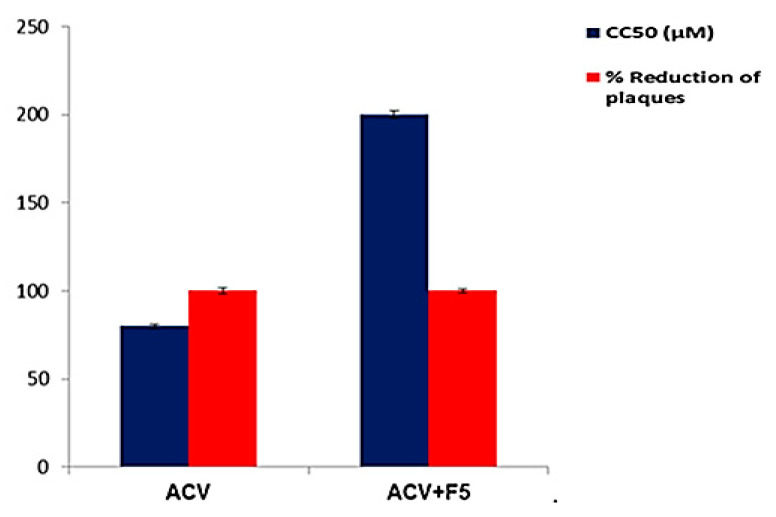
% Reduction of plaques and CC_50_ of acyclovir (ACV) before and after addition of F5 against HS-1 virus, each point represents the mean ± SD (n = 3). Abbreviation: F5, the optimized Curcumin-loaded proniosomal formula; ACV, Acyclovir; HS-1, Herpes simplex type I virus; CC_50,_ the half-maximal cytotoxic concentration.

Moreover, the Curcumin-loaded proniosomal formula (F5) was further tested for its combined effects with ACV on the anti-HSV-1 activity. F5 (30 μM) proved to increase the CC_50_ of ACV to 200 μM and reduce its effective dose that produced 100 ± 1.35% reduction of viral plaques from 12.2 μM to 6.25 μM (Figure 12). Moreover, F5 increased the SI value of ACV from 13.1 to 64.5. Accordingly, the safety and selectivity of ACV improved after the addition of F5. These findings showed the positive impact of adding the proniosomal Curcumin to ACV forming a potent and less toxic mixture.

According to the previous results, it is worth noting that the dry proniosomes are free-flowing powders that offer a more convenient method for preparing niosomes than other conventional techniques. Moreover, the in vitro release profile and stability of proniosomes are superior to the corresponding niosomes. Additionally, proniosomes of Curcumin have higher antiviral activity and safety than that of the corresponding Curcumin dispersion. Therefore, proniosomes are a promising drug delivery system for Curcumin.

## 4. Conclusions

The present study investigated the formulation of Curcumin-loaded provesicular nanocarriers as an effective antiviral agent. Nine Curcumin-loaded proniosomes were effectively prepared using the slurry method according to 3^2^ factorial design. The optimized proniosomal formulation (F5) was selected on the basis of maximizing both EE% and Q_12h_ according to the desirability criteria. F5 exhibited a prolonged release profile and relatively high EE%. Upon comparing with the corresponding niosomal formula, the optimized proniosomal formula demonstrated higher stability and a significant increase in Q_12h_. F5 exhibited a significant increase in both the antiviral activity and safety compared to pure Curcumin. Besides, the antiviral activity and safety of ACV improved after the addition of F5. In summary, these findings showed that Curcumin-loaded proniosomes are a promising and stable nanodelivery system that could overcome the pitfalls of the corresponding niosomes and improve both the antiviral activity and safety of Curcumin.

## Figures and Tables

**Table 1:** The prescreening study for formulation of Curcumin-loaded proniosomes.

Formula	Time of Hydration (min)	Volume of Hydration (mL)	* EE%
P1	5	10	94.11 ± 1.27
P2	5	20	82.13 ± 2.11
P3	30	10	94.23 ± 1.27
P4	30	20	82.30 ± 1.32

Notes: 1 g of maltodextrin per 1 mmole of the total lipid–surfactant mixture; * the values are presented as mean ± SD (n = 3). Abbreviations: EE, entrapment efficiency.

**Table 2:** Dependent and independent variables in 3^2^ factorial design used for the optimization of Curcumin-loaded proniosomes.

Formula Code	Variables				
	Independent		Dependent		
	X1	X2	Y1 *	Y2 *	
F1	−1	−1	89.06 ± 2.12	51.74 ± 1.23	
F2	−1	0	81.11 ± 1.36	76.56 ± 1.41	
F3	−1	1	68.61 ± 1.62	92.52 ± 2.69	
F4	0	−1	94.11 ± 1.27	42.71 ± 1.74	
F5 #	0	0	89.94 ± 2.31	70.89 ± 1.62	
F6	0	1	70.11 ± 1.49	89.76 ± 1.44	
F7	1	−1	81.89 ± 1.25	61.78 ± 1.38	
F8	1	0	69.17 ± 1.41	84.26 ± 1.34	
F9	1	1	60.33 ± 1.61	94.91 ± 2.18	
Independent variables			Low (−1)	Medium (0)	High (+1)
X1: Ratio of surfactant to CHOL			90:160	150:100	210:40
X2: Type of surfactant			Span 60	Span 60 & Tween 80	Tween 80

Notes: Y1: EE (%), Y2: Q_12h_ (%), * the data are expressed as mean ± SD (n = 3), # Optimized Formula, 1 g of maltodextrin per 1 mmole of the surfactant–lipid mixture. Abbreviations: Q_12h,_ drug released after 12 h; EE, entrapment efficiency; CHOL, cholesterol.

**Table 3:** Output data of the 3^2^ factorial design of Curcumin-loaded proniosomes.

Responses	R^2^	Adjusted R^2^	Predicted R^2^	Adequate Precision
EE% (Y1)	0.9676	0.9352	0.8359	16.372
Q_12h_ (Y2)	0.9822	0.9643	0.9097	20.165

Abbreviations: R^2^, the coefficient of determination; Q_12h_, % drug released after 12 h; EE, entrapment efficiency.

**Table 4:** ANOVA for the 3^2^ factorial design of Curcumin-loaded proniosomes.

Depndent Variable	Source	SS	DF	MS	F-Value	*p*-Value
Y1	Model	1053.93	4	263.48	29.84	0.0031
	X1	312.89	2	156.45	17.72	0.0103
	X2	471.03	2	370.52	41.97	0.0021
Y2	Model	2724.45	4	681.11	55.09	0.0009
	X1	235.90	2	117.95	9.54	0.0300
	X2	2488.55	2	1244.28	100.64	0.0004

Notes: Y1: EE (%), Y2: Q_12h_ (%), ratio of surfactant to CHOL (X1), the surfactant type (X2), *p*-value less than 0.05 shows that the model terms are significant. Abbreviation: SS, sum of squares; DF, degree of freedom; MS, mean of squares.

**Table 5:** The calculated correlation coefficients for the in vitro release of Curcumin and Curcumin-loaded proniosomes employing different kinetic orders.

Formula	Zero Order	First Order	Higuchi Model	Hixson Crowell	Korsmeyer-Pappas
F1	0.9890	−0.9958	0.9991	0.9940	0.9988
F2	0.9887	−0.9948	0.9978	0.9958	0.9924
F3	0.9902	−0.9839	0.9997	0.9964	0.9889
F4	0.9852	−0.9913	0.9957	0.9896	0.9952
F5	0.9854	−0.9952	0.9966	0.9939	0.9949
F6	0.9927	−0.9846	0.9993	0.9955	0.9860
F7	0.9842	−0.9948	0.9966	0.9925	0.9960
F8	0.9928	−0.9959	0.9998	0.9992	0.9948
F9	0.9931	−0.9663	0.9990	0.9890	0.9801
Curcumin	0.9903	−0.9897	0.9989	0.9982	0.9927

**Table 6:** Effect of storage on the properties of the optimized proniosomal formula and the corresponding niosomal formula.

Parameter	Proniosomal Formula		Niosomal Formula	
	Fresh	Stored	Fresh	Stored
Drug Content (%)	97.44 ± 1.69	96.95 ± 1.32	95.94 ± 1.27	90.22 ± 1.44
EE (%)	89.94 ± 2.31	87.86 ± 1.82	90.78 ± 1.52	83.02 ± 2.11
Q_12h_ (%)	70.89 ± 1.62	68.94 ± 0.90	61.42 ± 1.71	53.12 ± 0.97

Notes: Each value is presented as mean ± SD (n = 3), the optimized Curcumin-loaded proniosomes is F5. Abbreviations: EE, entrapment efficiency; Q_12h_, % drug released after 12 h.

## Supplementary Materials

The following are available online, Figure S1: FTIR spectrum of (a) Curcumin, (b) Tween 80, (c) Span 60, (d) maltodextrin, (e) CHOL, (f) plain proniosomes and (g) the optimized Curcumin proniosomal formula, Figure S2: DSC thermogram of (a) Curcumin, (b) Tween 80, (c) Span 60, (d) maltodextrin, (e) CHOL, (f) plain proniosomes and (g) the optimized Curcumin proniosomal formula, and Figure S3: (A) 3D presentation of DNA polymerase/Curcumin complex (B) Curcumin (Red) docked in the active site of DNA polymerase, (C) 2D presentation showing interaction of Curcumin with the active site.
