# Supplementary material for: Development of Provesicular Nanodelivery System of Curcumin as a Safe and Effective Antiviral Agent: Statistical Optimization, In Vitro Characterization, and Antiviral Effectiveness
Source: Molecules. 2020 Dec 1;25(23):5668. doi: 10.3390/molecules25235668 (PMC7731007; doi:10.3390/molecules25235668)
Supplement: Supplementary file 1 [file molecules-25-05668-s001.pdf]

Supplementary information

Fourier transform infrared spectroscopy (FTIR)

FTIR spectra of Curcumin, the chosen surfactant, plain proniosomes, and the optimized proniosomal formula were examined using the FTIR spectrometer (FT-IR Shimadzu 8300 Japan). KBr pellets were prepared in a hydraulic press (Kimaya Engineers, Maharastra, India) by mixing different samples (3 mg) with potassium bromide[1,2]. The range of scanning was 4000–400 cm^{-1}.

Figure S1 demonstrates the FTIR spectra of Curcumin, Tween 80, Span 60, CHOL, maltodextrin, plain proniosomes and the optimized proniosomal formula (F5). The IR spectrum of Curcumin demonstrated stretching vibrations due to phenolic hydroxyl groups at 3200–3500 cm^{-1}, stretching vibration at 1490 cm^{-1} associated with the aromatic C=C bond and a bending vibration at 1246 cm^{-1} attributed to the phenolic C-O group[3]. The FTIR spectrum of Tween 80 showed asymmetric and symmetric stretching bands of ($-CH_2$) at 2907 and 2855 cm^{-1}, respectively, a stretching band at 1735 cm^{-1} due to the C=O ester group and a strong band at 3436 cm^{-1} associated with the hydroxyl stretching vibrations[4]. Span 60 exhibited characteristic peaks at 3410 cm^{-1} due to aliphatic O–H stretch, 2936 cm^{-1} attributed to C–H stretch and 1745 cm^{-1} due to carbonyl stretch of ester [5]. The IR spectrum of maltodextrin investigated an absorption band at 3399 cm^{-1} due to the hydroxyl group, a broad band at 980 –1200 cm^{-1} which is a characteristic band for polysaccharides, and characteristic stretching bands at 1152 cm^{-1} and 1018 cm^{-1} due to the carbonyl group[6]. CHOL exhibited characteristic band assignments at 3400 cm^{-1} due to OH stretching and characteristic bands between 2800–3000 cm^{-1} attributed to stretching vibrations (asymmetric and symmetric) of CH_2 and CH_3 groups[5]. The FTIR spectrum of plain (drug-free) proniosomes demonstrated the characteristic peaks of CHOL, Tween 80, Span 60 and maltodextrin with a reduced intensity which may be explained on the basis of the development of lipid bilayer in the plain proniosomes[2]. The IR spectrum of the optimized Curcumin-loaded proniosomal formula (F5) showed the characteristics peaks of both Curcumin and different excipients with a minor shifting and decreased intensity. These findings investigated the absence of interactions between Curcumin and different excipients. The reduced intensity and minor shifting of the characteristic peaks of Curcumin might be attributed to the presence of some bonds, such as Van der Wall forces, hydrogen bond, or dipole interactions, between Curcumin and other excipients that results in increasing the entrapment of Curcumin within proniosomes[2].

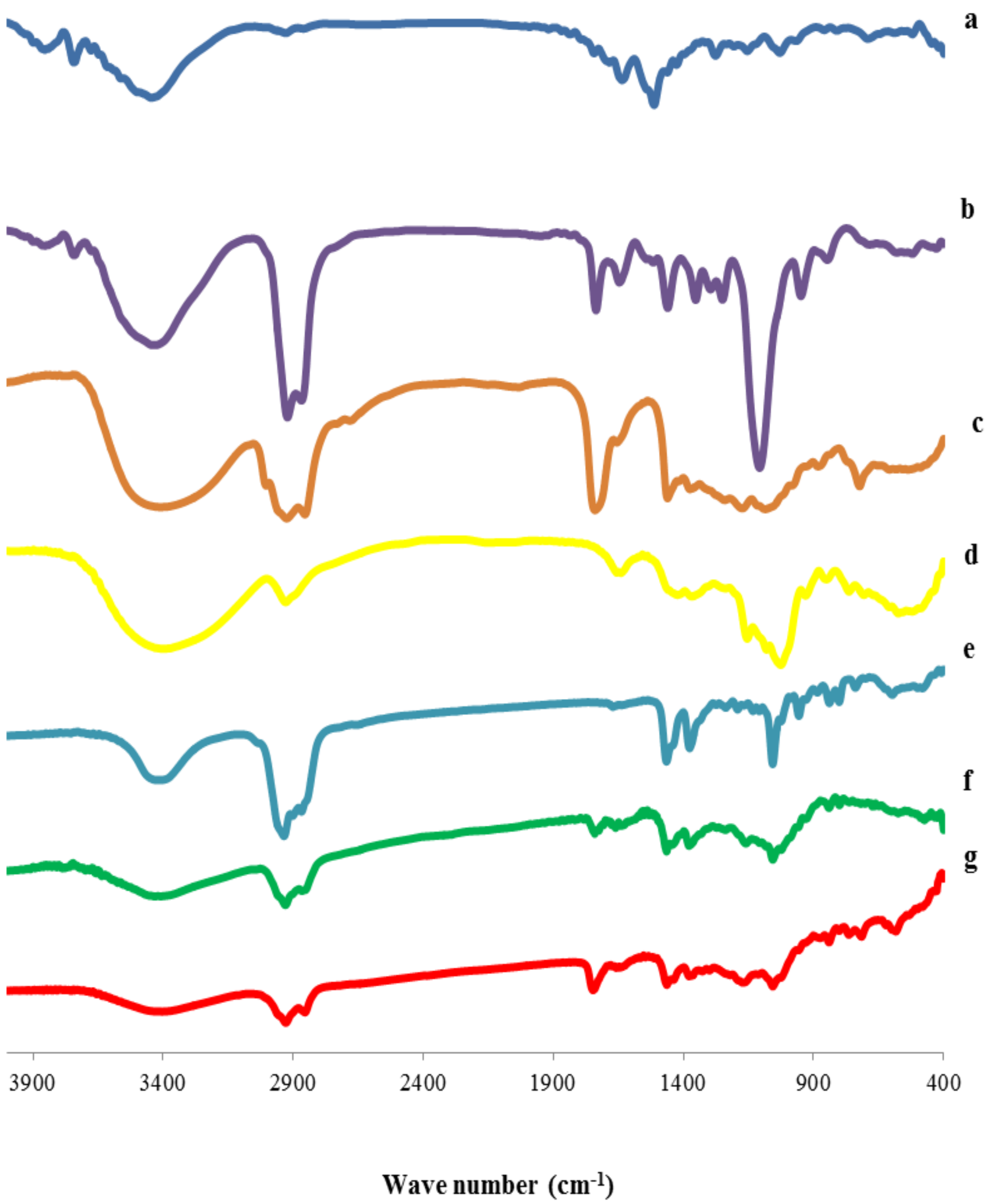

Figure S1. FTIR spectrum of (a) Curcumin, (b) Tween 80, (c) Span 60, (d) maltodextrin, (e) CHOL, (f) plain proniosomes and (g) the optimized Curcumin proniosomal formula.

Differential scanning calorimetry (DSC) study

DSC study was carried out using a Shimadzu DSC 60 (Japan, Kyoto). 3 mg samples of Curcumin, the chosen surfactant, plain proniosomes, and the chosen proniosomal formula were placed in aluminum pans and sealed. Thermograms were obtained by heating the samples from 20 to 260°C with a scan rate of 10°C/ min[2,7].

Figure S2 displays the DSC thermograms of Curcumin, Tween 80, Span 60, CHOL, maltodextrin, plain proniosomes and the optimized proniosomal formula (F5). The DSC thermogram of Curcumin reflected the crystallinity of Curcumin in its pure form. It showed a

characteristic endothermic peak at 179.8 °C which is corresponding to its melting point[8]. The DSC thermogram of Tween 80 exhibited an endothermic peak at 113.9°C that is attributed to its flash point[9,10]. The thermograms of Span 60, CHOL and maltodextrin demonstrated endothermic peaks at 53.5°C, 143.8°C and 293.7 °C, respectively due to their transition temperatures[11,12]. The plain proniosomes exhibited a broad endothermic peak at 197.2°C. The optimized Curcumin-loaded proniosomal formula (F5) demonstrated the endothermic peak of the lipid bilayer at 220.3°C with the absence of the characteristic melting peak of Curcumin. That could be explained on the basis of changing the structure of Curcumin from crystalline to amorphous state due to reasonable entrapment of Curcumin within proniosomes[13].

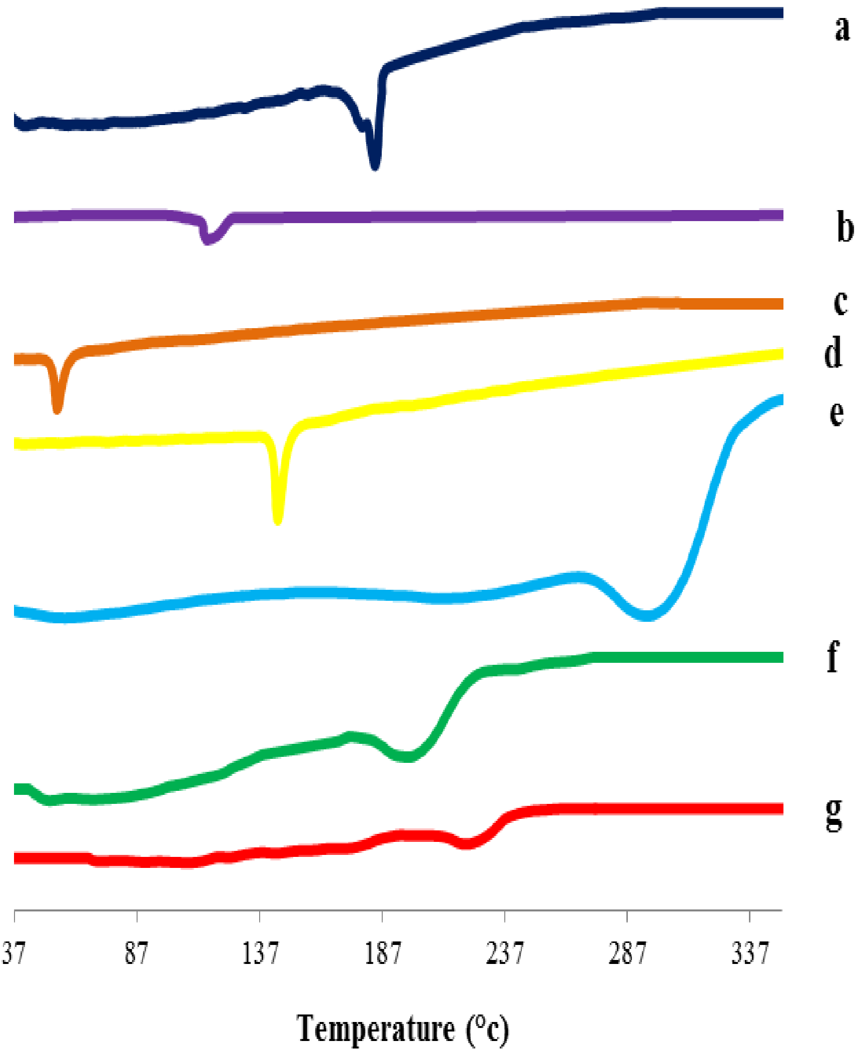

Figure S2. DSC thermogram of (a) Curcumin, (b) Tween 80, (c) Span 60, (d) maltodextrin, (e) CHOL, (f) plain proniosomes and (g) the optimized Curcumin proniosomal formula.

Molecular docking study

DNA polymerase is a critical enzyme in the lytic phase of the viral infection by HS viruses and vital for viral replication[14]. In order to investigate the ability of Curcumin to bind with DNA polymerase of HS-1, the 3D structures were obtained from PDB using the codes: 2GV9. The retrieved 3D structure was prepared using a quick prep module in molecular operating environment software (MOE), where water molecules were removed,

bond orders were assigned, hydrogen atoms were added, hydrogen bonds were optimized, charges were corrected, and the protein complex was minimized. The prepared PDB file of the protein was loaded in protein preparation module integrated into PyRX software for virtual screening[15] where it converted to Pdbqt file and the active site was defined according to Hu et al[16] where the grid box size was 25 x25x 25 and the coordinate was X: 28.2963, Y: -28.0432 and Z: 25.30. 3D structure of Curcumin was downloaded as mol2 file form zinc database[17], loaded to the ligands preparation module integrated in PyRX and converted to Pdbqt.

The molecular docking was proceeded using Autodock vina as the docking engine where exhaustiveness was set as 12 and the number of poses was 10, the software ranked the poses according to their binding energy(ΔG), and the docked poses was subjected for analysis to determine how they interacted with amino acid residues in the active site using LIGPlot Plus which generate a 2D presentation of complexes of the docked poses and proteins, where direct bonds are showed as dashed lines while hydrophobic interactions are showed as spline sections[18]. Predicted Ki was calculated based on the following equation: $\Delta G = -RT \ln (Ki)$[19].

The docking study involves predicting the ligand conformation and posing (or orientation) within a targeted binding site[20]. Virtual screening can be a useful tool for prediction and recognition of the pharmacological activities of drugs [21,22]. The viral DNA polymerase is a critical enzyme in the lytic phase of the viral infection by HS viruses[14]. The molecular docking study revealed the ability of Curcumin to bind effectively with the active site of DNA polymerase of HSV-1 achieving ΔG=-8.3 (kcal/mol) and estimated Ki=0.8 μM. That could be explained by its interaction with some key amino acids such as Cys371 and Lys539 by hydrogen bonding, and Leu379, Phe381, Phe382, Phe470, Tyr557, Tyr465, Asn466 and Gln978 through Van der Waals forces as shown in Figure S3 in addition to the ability of Curcumin to inhibit other molecular targets of HSV-1 such as thymidine kinase[23]. These findings could explain and predict the antiviral activity of Curcumin against HSV-1 virus.

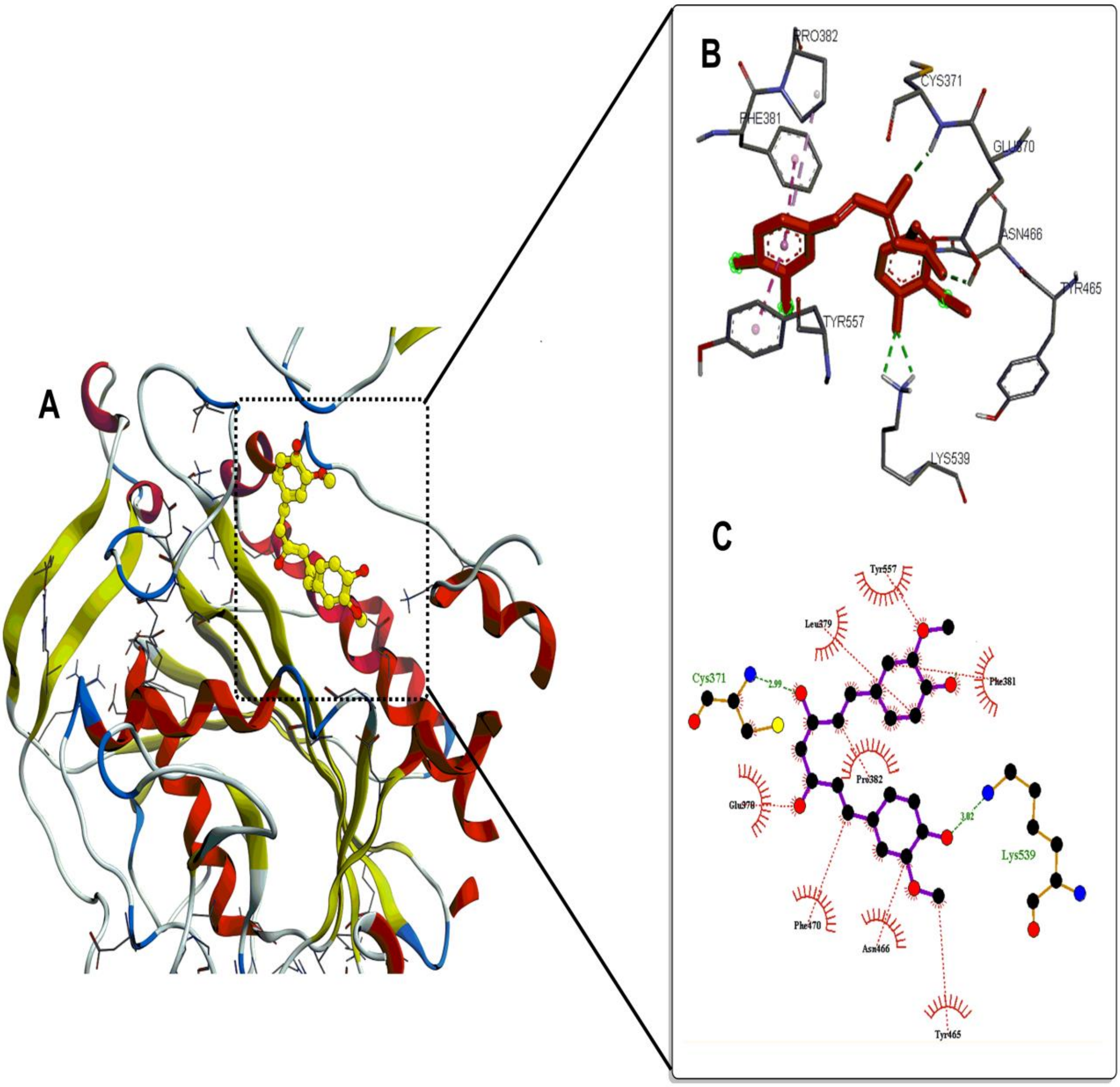

Figure S3. A) 3D presentation of DNA polymerase/Curcumin complex B) Curcumin (Red) docked in the active site of DNA polymerase, C) 2D presentation showing interaction of Curcumin with the active site.

References

1. Alemi, A.; Reza, J.Z.; Haghiralsadat, F.; Jaliani, H.Z.; Karamallah, M.H.; Hosseini, S.A.; Karamallah, S.H. Paclitaxel and curcumin coadministration in novel cationic PEGylated niosomal formulations exhibit enhanced synergistic antitumor efficacy. *Journal of nanobiotechnology* **2018**, *16*, 28.
2. Mazyed, E.A.; Zakaria, S. Enhancement of dissolution characteristics of clopidogrel bisulphate by proniosomes. *Int J Appl Pharm* **2019**, *11*, 77-85, doi:10.22159/ijap.2019v11i2.30575.
3. Ismail, E.; Sabry, D.; Mahdy, H.; Khalil, M. Synthesis and Characterization of some Ternary Metal Complexes of Curcumin with 1, 10-phenanthroline and their Anticancer Applications. *Journal of Scientific Research* **2014**, *6*, 509-519.
4. Ren, W.; Tian, G.; Jian, S.; Gu, Z.; Zhou, L.; Yan, L.; Jin, S.; Yin, W.; Zhao, Y. TWEEN coated NaYF 4: Yb, Er/NaYF4 core/shell upconversion nanoparticles for bioimaging and drug delivery. *Rsc Advances* **2012**, *2*, 7037-7041.
5. El-Sayed, M.M.; Hussein, A.K.; Sarhan, H.A.; Mansour, H.F. Flurbiprofen-loaded niosomes-in-gel system improves the ocular bioavailability of flurbiprofen in the aqueous humor. *Drug Dev. Ind. Pharm.* **2017**, *43*, 902-910.
6. Sun, P.; Yang, H.-J.; Wang, Y.-Q.; Liu, K.-Z.; Xu, Y.-W. Lipase-catalyzed synthesis and characterization of stearic acid dextrin ester. *Research in Health and Nutrition* **2013**, *1*, 7-11.
7. Sadeghi Ghadi, Z.; Ebrahimnejad, P. Curcumin entrapped hyaluronan containing niosomes: preparation, characterisation and in vitro/in vivo evaluation. *J. Microencapsul.* **2019**, *36*, 169-179.
8. Chopra, M.; Jain, R.; Dewangan, A.K.; Varkey, S.; Mazumder, S. Design of curcumin loaded polymeric nanoparticles-optimization, formulation and characterization. *Journal of Nanoscience and Nanotechnology* **2016**, *16*, 9432-9442.
9. Fang, J.-Y.; Fang, C.-L.; Liu, C.-H.; Su, Y.-H. Lipid nanoparticles as vehicles for topical psoralen delivery: solid lipid nanoparticles (SLN) versus nanostructured lipid carriers (NLC). *Eur. J. Pharm. Biopharm.* **2008**, *70*, 633-640.
10. Pramod, K.; Suneesh, C.V.; Shanavas, S.; Ansari, S.H.; Ali, J. Unveiling the compatibility of eugenol with formulation excipients by systematic drug-excipient compatibility studies. *J Anal Sci Technol* **2015**, *6*, 34.
11. Rahman, S.A.; Abdelmalak, N.S.; Badawi, A.; Elbayoumy, T.; Sabry, N.; Ramly, A.E. Formulation of tretinoin-loaded topical proniosomes for treatment of acne: in-vitro characterization, skin irritation test and comparative clinical study. *Drug deliv* **2015**, *22*, 731-739.
12. Das, S. Study of decomposition behaviour of binders and the effect of binder type on strength and density of alumina samples. 2011.
13. Patil, H.N.; Hardikar, S.R.; Bhosale, A.V. Formulation development and evaluation of proniosomal gel of carvedilol. *International journal of pharmacy and pharmaceutical sciences* **2012**, *4*, 191-197.
14. Zarrouk, K.; Piret, J.; Boivin, G. Herpesvirus DNA polymerases: structures, functions and inhibitors. *Virus Res.* **2017**, *234*, 177-192.
15. Dallakyan, S.; Olson, A.J. Small-molecule library screening by docking with PyRx. In *Chemical biology*, Springer: 2015; pp. 243-250.

16. Hu, L.; Zhang, Y.; Zhu, H.; Liu, J.; Li, H.; Li, X.-N.; Sun, W.; Zeng, J.; Xue, Y.; Zhang, Y. Filicinic Acid Based Meroterpenoids with Anti-Epstein–Barr Virus Activities from Hypericum japonicum. *Organic Letters* **2016**, *18*, 2272-2275, doi:10.1021/acs.orglett.6b00906.
17. Sterling, T.; Irwin, J.J. ZINC 15–ligand discovery for everyone. *Journal of chemical information and modeling* **2015**, *55*, 2324-2337.
18. Laskowski, R.A.; Swindells, M.B. LigPlot+: multiple ligand–protein interaction diagrams for drug discovery. ACS Publications: 2011.
19. Fukunishi, Y.; Yamasaki, S.; Yasumatsu, I.; Takeuchi, K.; Kurosawa, T.; Nakamura, H. Quantitative Structure-activity Relationship (QSAR) Models for Docking Score Correction. *Molecular informatics* **2017**, *36*, 1600013.
20. Kitchen, D.B.; Decornez, H.; Furr, J.R.; Bajorath, J. Docking and scoring in virtual screening for drug discovery: methods and applications. *Nature reviews Drug discovery* **2004**, *3*, 935-949.
21. Elgazar, A.A.; Knany, H.R.; Ali, M.S. Insights on the molecular mechanism of anti-inflammatory effect of formula from Islamic traditional medicine: An in-silico study. *Journal of Traditional and Complementary Medicine* **2019**, *9*, 353-363, doi:https://doi.org/10.1016/j.jtcme.2018.09.004.
22. Rollinger, J.M.; Stuppner, H.; Langer, T. Virtual screening for the discovery of bioactive natural products. In *Natural compounds as drugs Volume I*, Springer: 2008; pp. 211-249.
23. El-Halim, S.M.A.; Mamdouh, M.A.; El-Haddad, A.E.; Soliman, S.M.J.S.P. Fabrication of Anti-HSV-1 Curcumin Stabilized Nanostructured Proniosomal Gel: Molecular Docking Studies on Thymidine Kinase Proteins. **2020**, *88*, 9.
